# Questioning the Resistance/Aerobic Training Dichotomy: A commentary on physiological adaptations determined by effort rather than exercise modality

**DOI:** 10.2478/hukin-2014-0119

**Published:** 2014-12-30

**Authors:** James Fisher, James Steele

**Affiliations:** 1 Centre for Health Exercise and Sport Science, Southampton Solent University, East Park Terrace, Southampton UK.

**Keywords:** resistance training, exercise, cardiovascular, endurance, hypertrophy, strength

## Abstract

This paper discusses and challenges the current opinion that exercise adaptation is generally defined by modality; resistance exercise (RE), or aerobic exercise (AE). In presenting a strong body of recent research which demonstrably challenges these perceptions we suggest alternate hypotheses towards physiological adaptation which is hinged more upon the effort than the exercise modality. Practical implications of this interpretation of exercise adaptation might effect change in exercise adherence since existing barriers to exercise of time, costs, specialized equipment, etc. become nullified. In presenting the evidence herein we suggest that lay persons wishing to attain the health and fitness (including strength and muscle hypertrophy) benefits of exercise can choose from a wide range of potential exercise modalities so long as the effort is high. Future research should consider this hypothesis by directly comparing RE and AE for acute responses and chronic adaptations.

## Introduction

The purpose of this short communication is to promote open discussion of a topical issue within exercise physiology; the prevailing paradigms regarding specificity of adaptations to resistance exercise (RE) and aerobic/cardiovascular exercise (AE) and in addition, the potential implications of questioning them. For decades the exercise science and general communities have identified and differentiated between RE and AE, notably focusing on apparently divergent adaptations between exercise modalities. However, recently it has been suggested that performing RE to momentary muscular failure (MMF), and thus using a maximal effort, facilitates similar acute physiological responses and chronic physiological adaptations to traditional AE (Steele et al., 2012). Though this does not imply that performance of RE alone will result in optimal endurance activity performance, the physiological responses and adaptations independently appear similar to traditional AE. This signifies a paradigm shift from the historical thinking which typically dichotomises the two. In a similar vein, research has also suggested that effort is important in optimising adaptations to AE and that training above the lactate threshold produces significant gains in traditional AE performance beyond that of AE utilising lower effort levels (Henritze et al., 1985; Wletman et al., 1992). The notion that both RE and AE at higher physiological effort levels produce greater aerobic/cardiovascular adaptations is becoming more widely accepted. However, we might also consider the contrasting perspective as to whether traditional AE modalities can also produce physiological adaptations similar to that of RE i.e. muscular strength and hypertrophy. A recent review has also questioned the historical dichotomy between RE and AE in this respect presenting evidence that traditional AE holds the potential to induce hypertrophic adaptations in a range of populations similar to RE (Knopka and Harber, 2014). Whilst comparison of magnitude of adaptations is not possible due to the lack of research, that RE and AE can produce seemingly similar physiological adaptations suggests the need to alter the typical paradigm under which exercise scientists have laboured for some time.

## Effort, Energy Systems, Fatigue and Failure

It is perhaps worthwhile revisiting our existing terminology for RE and AE regarding exercise *‘intensity’*. In the AE literature intensity is considered a measure of physiological exertion, whilst in RE intensity is often used to denote load (e.g. %1RM). Previous publications have discussed the use and potential misuse of these terms (e.g. Fisher and Smith, 2012; Steele, 2013) ultimately suggesting that intensity refers to the magnitude of a particular measure and that terminology should denote what it is a measure of (e.g. intensity *of* effort, intensity *of* load). To avoid confusion when discussing physiological exertion (muscular, cardiovascular and cardiorespiratory) for both RE and AE in the present piece we elected simply to use the term *effort*. In this sense it seems appropriate to consider effort in terms of metabolic demands. For example, sustained low effort is aerobic, but as effort increases beyond the threshold the body transitions towards anaerobic energy production where metabolites are accumulated potentially limiting the duration of exercise at this increased effort level. In this sense high effort is generally a reference to anaerobic exercise. With this in mind we should consider that cycling, running, swimming etc. (traditionally considered AE modalities) can be either predominantly aerobic or anaerobic depending on effort influencing bioenergetic pathways, whilst RE (typically anaerobic) if performed at a low enough effort level can be aerobic. Of course this adds further confusion to the traditional comparison of RE and traditional AE in the sense that AE is not necessarily aerobic. For the purposes of the present article AE refers to modalities of exercise traditionally thought of as aerobic, e.g. cycling, running, swimming, etc. and not the effort level.

It is also worth discussing effort in relation to muscular failure and fatigue. Edwards (1983) defined fatigue as *“failure to maintain the required or expected power output”*. Fitts (1994) commented that this definition accommodated both force and velocity, as such this was fitting for both AE and RE. However, exercise scientists often make reference to *failure,* both within RE and AE, as the point of cessation of exercise because of an inability to meet the external demands of the exercise. It may therefore be more appropriate to consider *fatigue* as an ongoing process whereby a person’s ability to produce muscular force decreases progressively in magnitude as they continue exercise, with a concomitant progressive increase in *effort* required, that ultimately culminates in *failure* to match the external demands of the exercise. Fitts’ (1994) discussion of the determinants of fatigue in relation to short-duration high-effort and prolonged submaximal exercise perhaps fits this conceptualisation. Whilst it is beyond the scope of this commentary to elaborate, in brief Fitts described fatigue to involve the *“recruitment of all three fibre types, a high contraction frequency and a high degree of anaerobic metabolism”* continuing that the *“high level of anaerobic metabolism will lead to an increase in intracellular H ^+^* (Hydrogen ions) *and Pi* (inorganic Phosphate)*, factors known to inhibit peak force”*. As such AE and RE when performed at sufficiently high effort level will use the same metabolic pathways and thus catalyse the same fatigue responses.

## Recent research

A number of recent studies have led to the above questioning of the RE/AE dichotomy. It has been recommended that RE performed to an effort which sequentially and maximally recruits as many motor units and muscle fibres as possible (e.g. MMF; Carpinelli, 2008; Jungblut, 2009) stimulates the most significant strength (Fisher et al., 2011) and hypertrophic gains (Fisher et al., 2013). Further, recent evidence has suggested that maximally recruiting muscle fibres in a cycle task (typically considered AE) to muscular failure can also result in significant hypertrophy (Lundberg et al., 2013). Lundberg et al. required participants to perform a unilateral cycle task at 70% of maximal power (70%Wmax) at a cadence of 60 rpm for 40 minutes after which *“…the workload was increased by ∼20W, and subjects were requested to continue until failure”*. This progression in the required effort is critical to the current thinking. Lundberg et al. (2013) defined this modality to be aerobic exercise (AE), which might be a product of either easy definition for the comparison to RE within their study, or a result of the historical thinking that a prolonged cycle task is predominantly aerobic. This however, is irrespective of the fact that participants cycled until *failure* which as highlighted suggests the task to be anaerobic in nature, at least at cessation of exercise. The authors also reported that they recorded central and localised ratings of perceived exertion (RPE) noting higher values for localised RPE which reached maximal values at AE completion. The results of the study showed that a group performing AE+RE attained significantly greater hypertrophy than a group performing RE only (14% vs. 8%, respectively; *p* <0.05). In their discussion the authors commented *“Although we acknowledge these collective findings remain controversial, it may be that low-force actions repeated until failure ultimately promote muscle hypertrophy”.* However, we disagree that these findings should represent any form of controversy.

Recent research supports that similar gains in muscle hypertrophy are attainable by heavy or light external loads when RE is continued to a point of muscular failure (Mitchell et al., 2012; Ogasawara et al., 2013; Van Roie et al., 2013). Indeed, there appears little evidence that the use of heavy loads produces significantly greater gains in hypertrophy than training with lighter loads (Fisher et al., 2013; Steele et al., 2014). The use of different external resistance *types* (i.e. free weights, machines etc.) during RE also does not appear to yield any differences in either strength (Fisher et al., 2011) or hypertrophy (Fisher et al., 2013). In fact, it has been speculated based upon these findings that a muscle cannot identify the external resistance mode it is contracting against (whether that be free-weight, resistance machine, or cycle ergometer); a muscle either contracts or relaxes (Fisher et al., 2011). Indeed a recent study has demonstrated that external resistance may not even be a requirement for inducing hypertrophy. Maeo et al. (2013) reported that use of isometric co-contractions involving maximal voluntary contractions of antagonistic muscle groups against one another can induce significant strength and hypertrophic adaptation. Furthermore, previous research has also evidenced the potential hypertrophic gains from extended cycle tasks at a high relative effort, even when not performed to muscular failure (Harber et al., 2009). Ultimately, this body of evidence supports that *low-force repetitions to failure* (or to a sufficiently high effort), irrespective of the exercise modality and thus external resistance, promote muscular hypertrophy.

In support, whilst reviewing the mechanisms, Schoenfeld (2010) concludes that metabolic stress is likely a requirement for stimulating muscular hypertrophy. Whilst Schoenfeld also discusses other potential mechanisms which are contentious in the literature (e.g. hormonal responses in testosterone and insulin like growth-factor, along with frequency, load, repetitions, volume and rest periods) he clarifies that at a fundamental level hypertrophy is a product of protein synthesis exceeding protein breakdown, and the activity of satellite cells when sufficient mechanical stimulus is imposed on skeletal muscle. Since a muscle appears to respond and adapt with little bias to external demands (i.e. exercise modality), it seems reasonable to suggest that metabolic and mechanical stress as a result of any sufficiently high effort exercise is sufficient to stimulate hypertrophy, as long as post exercise protein synthesis exceeds protein breakdown.

## Challenging current dogma

Previous publications have discussed this dichotomy between the modalities, with regard to health outcomes (Phillips and Winett, 2010), and have generally reported in favour of RE above AE due to the plethora of health benefits including but not limited to reduced risk of cardiovascular disease, increased resting metabolic rate, improved blood lipid profiles, reduced resting blood pressure, improved bone mineral density and pain reduction for those suffering from arthritis (Phillips and Winett, 2010). However, the recent interest and research considering low-volume, ‘high-intensity interval training’ using 30 s maximal effort cycle sprint tasks (traditionally perceived as AE) has also reported similar health benefits (Gibala et al., 2012). With this in mind, the relationship between physiological adaptations from RE or AE when both are performed to maximal effort appears closer than has historically been portrayed. The evidence suggests that when effort is maximal or near maximal, similar physiological adaptations promoting aerobic/cardiorespiratory fitness *AND* hypertrophy, along with the aforementioned health measures, are likely irrespective of the exercise modality. And more, that effort appears to be the single most significant controllable variable to determine physiological adaptations to exercise.

We should clarify that at present a lack of research prevents a robust conclusion in this regard. As such we present this hypothesis based upon recent research. In addition, we have not discussed muscle fiber type adaptations or magnitude of change between modalities which, once again future research will hopefully consider in more detail. It would also be imprudent not to highlight that the studies mentioned were short in duration (5–12 weeks) and consider generally recreationally active or untrained subjects where physiological adaptations of any kind are likely to be most significant. It might be unlikely that a cyclist or runner can attain the required extremes of cardiovascular fitness from RE, or that a bodybuilder might achieve the desired muscle hypertrophy from AE. Indeed, athletically committed persons engaging in long-term training might better attain the desired adaptations by pre-existing exercise methods. However, this once again presents scope for future research.

## Conclusion and Practical implications

The potential implications of questioning the existing paradigm are quite profound if found to be supported through further investigation. Numerous studies report the most commonly cited barriers to exercise participation include time availability as well as access to specialised equipment and/or facilities; such as travel time and costs etc. (Sallis et al., 2000; McCromack et al., 2004; Kimm et al., 2006; Daskapan et al., 2006; Gomez-Lopez et al., 2010). In light of the concept discussed herein, persons wishing to engage in exercise in order to improve the noted markers of health and fitness might be able to select from a wide range of potential exercise modalities in order to achieve this. The caveat being, regardless of the modality chosen, persons should aim to exercise to a high level of effort in order to maximise these benefits. Exercise utilising high effort and shorter duration addresses the potential barrier of time in exercise participation. Further, the notion that exercise modality may be inconsequential potentially addresses the perception that specialised equipment and/or facilities are required, thus opening up a range of possibilities for lay persons wishing to improve health, fitness and muscle size effectively.

Aside from the proposed paradigm shift and implications for recommendations to improve public exercise participation, this commentary aims to encourage future research directly comparing RE and AE for acute responses and chronic adaptations whilst appropriately controlling for effort. As cycling has been the mode typically examined until now, of particular interest are other traditional AE modalities such as running, rowing etc. Indeed, it might be that future research suggests it is time to re-label our modalities of exercise to better portray our effort/physiological energy systems and/or desired responses and adaptations.
